# Structural changes upon membrane insertion of the insecticidal pore-forming toxins produced by *Bacillus thuringiensis*

**DOI:** 10.3389/finsc.2023.1188891

**Published:** 2023-04-26

**Authors:** Sabino Pacheco, Isabel Gómez, Angel E. Peláez-Aguilar, Luis A. Verduzco-Rosas, Rosalina García-Suárez, Nathaly A. do Nascimento, Lucero Y. Rivera-Nájera, Pablo Emiliano Cantón, Mario Soberón, Alejandra Bravo

**Affiliations:** Departamento de Microbiología Molecular, Instituto de Biotecnología, Universidad Nacional Autónoma de México, Cuernavaca, Morelos, Mexico

**Keywords:** *Bacillus thuringiensis*, pore-forming activity, Cry toxin, Vip3 toxin, Tc toxin

## Abstract

Different *Bacillus thuringiensis* (Bt) strains produce a broad variety of pore-forming toxins (PFTs) that show toxicity against insects and other invertebrates. Some of these insecticidal PFT proteins have been used successfully worldwide to control diverse insect crop pests. There are several studies focused on describing the mechanism of action of these toxins that have helped to improve their performance and to cope with the resistance evolved by different insects against some of these proteins. However, crucial information that is still missing is the structure of pores formed by some of these PFTs, such as the three-domain crystal (Cry) proteins, which are the most commercially used Bt toxins in the biological control of insect pests. In recent years, progress has been made on the identification of the structural changes that certain Bt insecticidal PFT proteins undergo upon membrane insertion. In this review, we describe the models that have been proposed for the membrane insertion of Cry toxins. We also review the recently published structures of the vegetative insecticidal proteins (Vips; e.g. Vip3) and the insecticidal toxin complex (Tc) in the membrane-inserted state. Although different Bt PFTs show different primary sequences, there are some similarities in the three-dimensional structures of Vips and Cry proteins. In addition, all PFTs described here must undergo major structural rearrangements to pass from a soluble form to a membrane-inserted state. It is proposed that, despite their structural differences, all PFTs undergo major structural rearrangements producing an extended α-helix, which plays a fundamental role in perforating their target membrane, resulting in the formation of the membrane pore required for their insecticidal activity.

## 1 Introduction

The production and use of pore-forming toxins (PFTs) is the main strategy used by several bacterial pathogens for the infection of their targets. PFTs must undergo significant conformational changes during their transition into membrane-inserted pores. Most of the reported PFTs are highly dynamic proteins as they change from their initial soluble monomeric conformations to final oligomeric, ring-like structures that are capable of membrane insertion. In these oligomeric arrangements, multiple monomers assemble together to form the oligomeric “pre-pore” that once located in the target membrane, undergoes further conformational changes to finally form the “functional pore” inserted into the membrane, that affects cell integrity and kills the target (1, 2). The transition from pre-pore to membrane-inserted pore structure may involve multiple rearrangements, including the reorganization of the hydrophobic core; transitions in secondary structures, such as transitions from loop regions or α-helices into β-sheets or β-sheets or loop regions into α-helices; and the formation of extended α-helices (3).

*Bacillus thuringiensis* (Bt) bacteria produce different insecticidal PFTs that kill insects, such as the insecticidal crystal (Cry) proteins, the vegetative insecticidal proteins (Vips; e.g., Vip3, Vpa1, and Vpa2), and the toxin complex (Tc), among others (4, 5). Bt PFTs break down the midgut epithelia cells of the larval stages of insects, representing a highly effective and interesting ecological strategy for pest control because they are both highly specific against target insects and biodegradable (6). Some of these PFT proteins have been successfully used worldwide to control different insect crop pests through their incorporation into sprayable formulations or expression in transgenic plants (7, 8). In addition, other Bt PFT proteins have been incorporated in formulations applied worldwide for the control of mosquito populations that are vectors of human diseases, such as malaria, dengue, Zika, and chikungunya (9). The high specificity of Cry and Vip3 proteins is based on their specific interactions with larval midgut membrane proteins, known as receptors (6).

Cry toxins are the most known and widely used proteins for the management of insect pests worldwide, and multiple studies have been reported describing their mechanism of action (6, 10, 11). However, the three-dimensional (3D) structure of the Cry oligomer required for pore-forming activity into the membrane has not yet been identified, information that is crucial for the future development of more effective Cry toxins that can affect novel targets or that can overcome insect resistance. Here we review the different proposed models for Cry membrane insertion.

In addition, we review the reported data showing that PFTs produced by Bt display important conformational changes during their transition from soluble proteins to membrane-inserted pores. For example, the 3D structures of Vip3Aa and Vip3Bc pre-pores and membrane-inserted pores were recently revealed through the use of cryo-electron microscopy analyses, which showed that both proteins exhibit similar conformational changes during their oligomerization and activation into the pore structures (12, 13). In this review, we will describe these conformational changes of the Vip3 proteins, which principally involve the N-terminal region that forms a long four-helical coiled-coil helix needle at the base of the oligomeric complex that is needed for insertion into the membrane and pore formation (12, 13).

Tcs are also insecticidal PFTs. The 3D structures of the Tc pre-pore and the membrane-inserted pore have been reported and are also discussed here. The Tc toxins are multi-subunit protein complexes consisting of three components: TcA, TcB, and TcC proteins. They were originally identified in different bacteria, such as *Photorhabdus luminescens* and *Xenorhabdus nematophila*, but genomic information from Bt showed that these genes are also present in different Bt strains. Briefly, the TcA proteins form a large pentameric structure and the TcB/TcC proteins are located at the top of this complex (14). A large conformational change in TcA proteins is required for pore formation, forming an inner helical needle that moves downward to penetrate the membrane forming the pore (14). In the final step, the C-terminal region of the TcC component is cleaved and translocated into the cytoplasm, where it shows adenosine diphosphate (ADP)-ribosyltransferase activity, leading to cell death (15).

The main objective of this work is to review the conformational changes of PFTs produced by Bt and to evaluate the different models that have been proposed to describe these conformational changes during their interactions with the insect target membrane.

## 2 Cry PFTs produced by Bt

**Figure 1:**
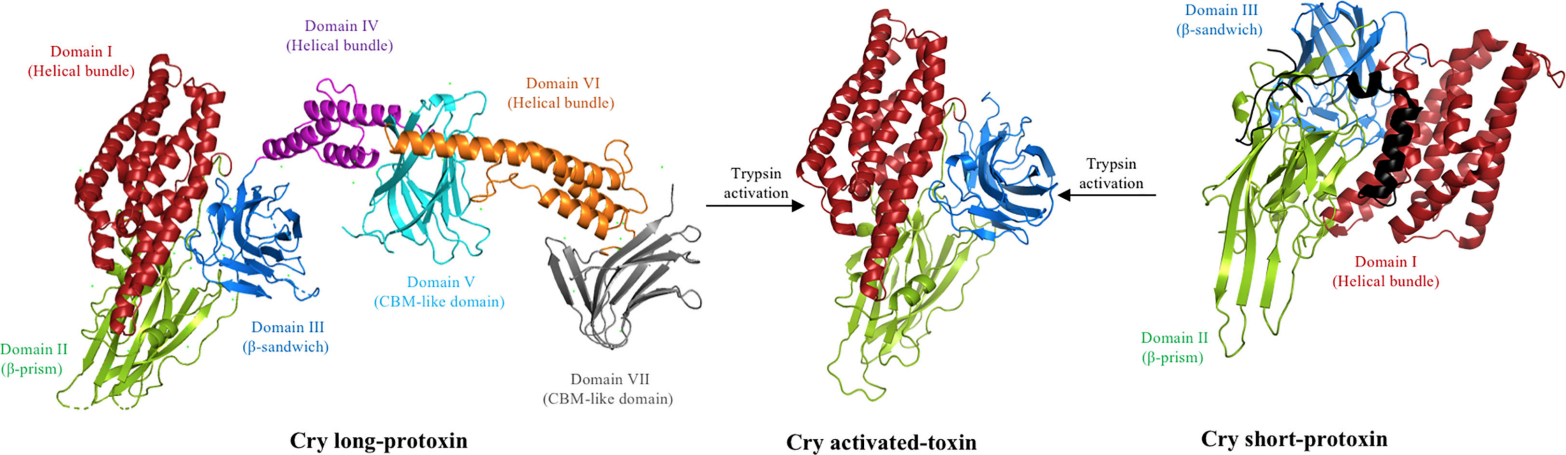
Proteolytic activation of the Cry toxin. Deposited 3D structure of a long Cry1Ac protoxin (PDB: 4W8J), a short Cry2Aa protoxin (PDB: 1I5P), and the trypsin-activated Cry1Aa toxin (PDB: 1CIY) were used to generate this figure by using the PyMol program. The trypsin-like proteases present in the insect midgut cleaves the long protoxin of 130 kDa, removing domains IV–VII to form an activated protein of approximately 60 kDa composed of three structural domains resistant to proteolysis. Note that the N-terminal region (approximately 30 residues) of the Cry1Ac protoxin is not shown and is also removed upon activation. In the case of the short 70-kDa protoxin, the N-terminal region (shown in black) is removed by the action of trypsin protease, resulting in a similar activated toxin composed of three structural domains resistant to proteolysis.

Cry toxins represent the largest protein family of Bt bacteria, with more than 755 members, and are classified into 57 different groups and 166 subgroups according to their amino acid sequence (5). These proteins are produced as protoxins, with a molecular mass of 130 or 70 kDa (**Figure 1**), and form crystal inclusions, also known as parasporal crystals. These parasporal crystals (with the exception of Cry1I protoxins, which are secreted into the medium) are formed inside the mother cell compartment during the bacterial sporulation phase (5).

As mentioned above, the 3D structures of the pre-pores and membrane pores of these toxins remain unknown. In this review, we will focus on the pore-forming activity of Cry toxins and the different models that have been utilized to identify their conformational changes upon membrane insertion. For this reason, we will not detail the Cry receptors that have been identified in multiple target insects and we recommend reviewing other reports that discuss this subject (10, 11, 16, 17).

When the parasporal crystals are ingested by susceptible larvae, they are solubilized inside the larval gut (10). The soluble protoxins are then able to bind to specific proteins, known as receptors, such as aminopeptidase N (APN) and alkaline phosphatase (ALP), located in the apical microvilli membrane of the midgut cells (18). Alternatively, the protoxins are cleaved by the midgut proteases into activated toxins with a mass of ≈ 60 kDa, that also bind to APN and ALP receptors and to other proteins, such as cadherin or adenosine triphosphate (ATP)-binding cassette (ABC) transporters (6, 18–20).

A previous study revealed the 3D structures of a long Cry1Ac (130 kDa) and short Cry2Aa protoxin (70 kDa), showing that the Cry1Ac protoxin consists of seven domains (21) (**Figure 1**), whereas the Cry2Aa protoxin has a three-domain organization (22). The 3D structural data of several activated Cry toxins showed a similar structure to the short protoxin, consisting of three domains, which also form the first three domains of the long protoxin (23–32) (**Figure 1**). Domain I contains seven α-helices and it is involved in oligomerization and membrane insertion, whereas domains II and III both consist mainly of β-sheets structures and are involved in receptor binding and specificity (6, 10). The 130-kDa protoxin contains four additional domains, and it is surprising that two of these domains (i.e., V and VII) are also composed principally of β-sheet structures that show some structural similarities to domains II and III of the activated-toxin, resembling carbohydrate-binding modules (21).

It was proposed that after receptor binding the Cry proteins form 150- to 250-kDa oligomers, and that the size of the oligomeric structure may depend on whether the protoxin or the activated toxin binds to the receptors (19, 33). The interactions with the cadherin receptor and ATP-binding cassette subfamily C member 2 (ABCC2) transporters are critical for oligomerization (19, 34). In contrast, incubation with other receptors, such as APN and ALP, did not induce Cry toxin oligomerization (19, 35). However, it has been proposed that these receptors participate in the insertion of Cry oligomers into the target membrane (19). These two different pre-pores can be distinguished by their size, heat sensitivity, and kinetics of pore formation in synthetic lipids, in which the 250-kDa pre-pore obtained from the protoxin is more heat-resistant than the 150-kDa pre-pore obtained from the activated toxin (19, 36). These oligomeric structures have been observed by scanning transmission electron microscopy (STEM) and atomic force microscopy (AFM), and their pore formation function has also been analyzed using different electrophysiological assays (19, 37–39).

### 2.1 Proposed models to explain the pore-forming activity of Cry toxins

#### 2.1.1 The “umbrella” model

**Figure 2:**
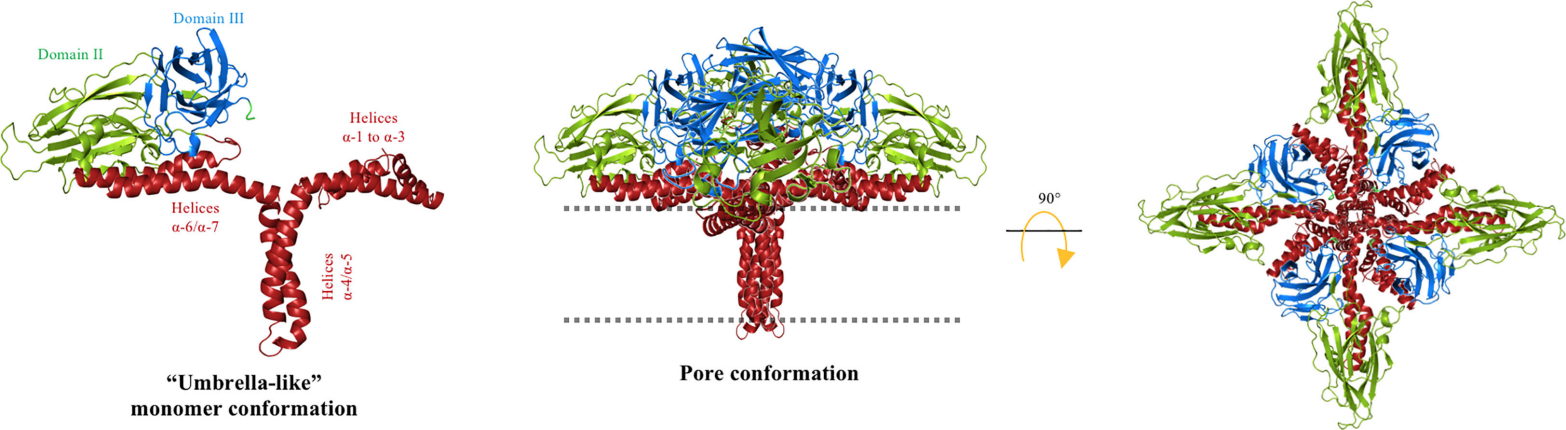
“Umbrella” model for Cry toxins. The helical domain I of the Cry protein is reordered to insert the hairpin α-4/α-5 into the cell membrane (dotted line) to form the lytic pore. The hydrophilic face of helix α-4 is facing the pore lumen and the hydrophobic helix α-5 is oriented at the lipid membrane. Helices α-1, α-2, α-3, α-6, and α-7 are partially embedded on the membrane surface, whereas domains II and III remain solvent exposed. A tetrameric conformation was proposed. The cell membrane is indicated by the dotted line. The figure was generated by using the PyMol program.

Since the 3D structure of Cry3Aa was explicated in 1991 (23), it has been proposed that the pore of Cry proteins may involve helix α-5, because this is a hydrophobic helix located in the center of the α-helices bundle that forms domain I (23). The “umbrella” model proposed that the hairpin formed by helices α-4 and α-5 is involved in insertion into the membrane, whereas the rest of the helices remain on the membrane surface (40). Further analysis performed with different Cry mutant proteins supported this model of toxin insertion (**Figure 2**), as mutations of charged residues from helix α-4, such as Cry1AaE129K, Cry1AaE129C, Cry1AaR131Q, Cry1AaR131D, Cry1AaR131E, Cry1AaR131H, or Cry1AaD136N, were severely affected in their toxicity against *Plutella xylostella* or *Manduca sexta* larvae (41, 42). In addition, the analysis of the pore-forming activity, by osmotic swelling assay (light-scattering assay), of these mutants in apical microvilli membranes (isolated from the midgut tissue of *M. sexta* larvae) showed that their pore-forming activity was affected, supporting the hypothesis that helix α-4 plays an important role in toxin action (41, 42). These data were confirmed by Cys-scanning mutagenesis, which demonstrated that several residues of helix α-4 were changed to cysteine, showing that mutations located on the hydrophilic face of the helix (i.e., Cry1AaR131C, Cry1AaQ133C, Cry1AaN135C, Cry1AaA140C, Cry1AaT142C, Cry1AaA144C, Cry1AaP146C, and Cry1AaL147C) severely affected both insecticidal and pore-forming activity (43). The negative charge of residue Asp-136 located in helix α-4, was proposed to be facing the lumen of the pore (**Figure 2**), as Cry1AaD136C mutant, was severely affected in pore-forming activity analyzed in black lipid bilayers, and assays in the presence of the negatively charged 2-sulfonatoethyl methanethiosulfonate sodium salt (MTSES) reagent that binds covalently to the sulfhydryl group of the Cys, reintroducing a negative charge in this position resulted in the complete recovery of pore-forming activity (41). Other studies indicated that the helix α-4 participates in oligomerization, as mutation Cry1AcN135Q affected oligomerization and toxicity against *M. sexta* (44). In addition, based on the Cry4Ba three-dimensional structure, a model simulating the insertion of the α-4–α-5 hairpin was constructed with the aim of increasing understanding of the pore’s possible structure (45).

However, additional data contradict this model, suggesting that the conformational changes observed during pore formation are more complex than the proposed movement of the α-4 and α-5 hairpins. For example, Cry1Aa mutants in the charged residues of the helix alpha-3 (i.e., Cry1AaR99E, Cry1AaR99Y Cry1AaE101C, Cry1AaE101Q, Cry1AaE101K, Cry1AaE116K, Cry1AaE118C, and Cry1AaD120K) were also affected in terms of toxicity against *M. sexta* and showed a complete lack of pore-forming activity, as analyzed in microvilli membranes from *M. sexta* (46), indicating that helix α-3 also plays an important role in pore formation. Similarly, mutations in Ala92 and Arg93 residues of Cry1A toxins, located at the beginning of helix α-3, reduced toxicity against *M. sexta* and affected pore-forming activity, as measured by their ability to disrupt K^+^-dependent amino acid transport in *M. sexta* microvilli membrane vesicles (47). It was proposed that helix α-3 participates in oligomerization, as Cry1AbR99E and Cry1AbY107E mutants were unable to form oligomers, which directly correlated with their lack of toxicity and pore-forming activity (48). In a different report, the simultaneous analysis of Förster resonance energy transfer (FRET) distances between specific sites in the Cry1Aa toxin and the membrane toxin (using synthetic lipid bilayer and electrical currents analysis) indicated that pore-forming activity was only observed after the movement of the hairpin composed of helices α-3 and α-4, which, in turn, suggested that the conformational change of this region is important for the pore-forming activity of Cry toxins (49). In this report, it was also shown that helix α-5 had a lack of movement, which contradicts the “umbrella” model (49). In contrast, Cry1Aa residues Ser39 and Phe50, located in helix α-1 and in the loop between helices α-2a and α-2b, respectively, were shown to be highly mobile, suggesting that these regions may also change their conformation during membrane insertion (49).

Finally, double Cys mutations were introduced into the Cry1Aa toxin to generate disulfide bridges to restrict movement between different α-helices during membrane insertion (bridging helices α-3 with α-4, R99C-A144C; α-5 with α-6, V162C-A207C; α-5 with α-7, S176C-S252C; and α-7 with domain II, R224C-S279C) (50). The analysis of such double mutations also contradicted the “umbrella” model, as mutants where helix α-5 was linked to helix α-6 or to helix α-7 were toxic to the larvae and showed pore-forming activity similar to that of wild-type proteins. The mutant that links helix α-7 to domain II was also toxic, indicating that the movement of helices α-4 to α-7 was not necessary for pore-forming activity. The only exception was the mutant with the disulfide bridge between helices α-3 and α-4, of which the toxicity and pore forming activity was altered, suggesting that movement of these helices was necessary of helix α-3 to helix α-4 was necessary for pore formation (50). However, it was shown that the single mutant Cry1AaR99C was also completely inactive (50), since, as mentioned above, this residue is implicated in oligomerization and pore-forming activity (48). It is interesting that a different report stated that all four double mutants lacked channel activity in the oxidized state and recovered pore-forming activity in the presence of the reducing agent β-mercaptoethanol when the pore formation assay was performed in the absence of Cry toxin receptors by analyzing pore formation in planar lipid bilayers constructed with synthetic lipids (51). Unfortunately, mutations involving other residues of helix α-3 or residues in helices α-1 and α-2 were not constructed, as the helices α-1, α-2a, and α-2b were too small to span the membrane bilayer (23, 24). Nevertheless, it was previously shown that the helix α-2 is broken by a highly conserved Pro residue located in the loop connecting helices α-2a and α-2b in the Cry toxin family. Interestingly, this residue’s mutations (i.e., Cry1AbP70A and Cry1AbP70G) decreased ion transport ability and reduced toxicity (52).

### 2.2 The “buried dragon” model

Protease protection assays of Cry1Ac toxin, performed with proteinase K showed that when the toxin is inserted into the membrane, the complete protein, with the exception of helix α-1, is protected from digestion, suggesting that the whole toxin is buried into the membrane (53). However, one possible explanation of these data is that Cry toxins display a compact structure, whereas helix α-1 is highly flexible and thus more susceptible to protease degradation.

**Figure 3:**
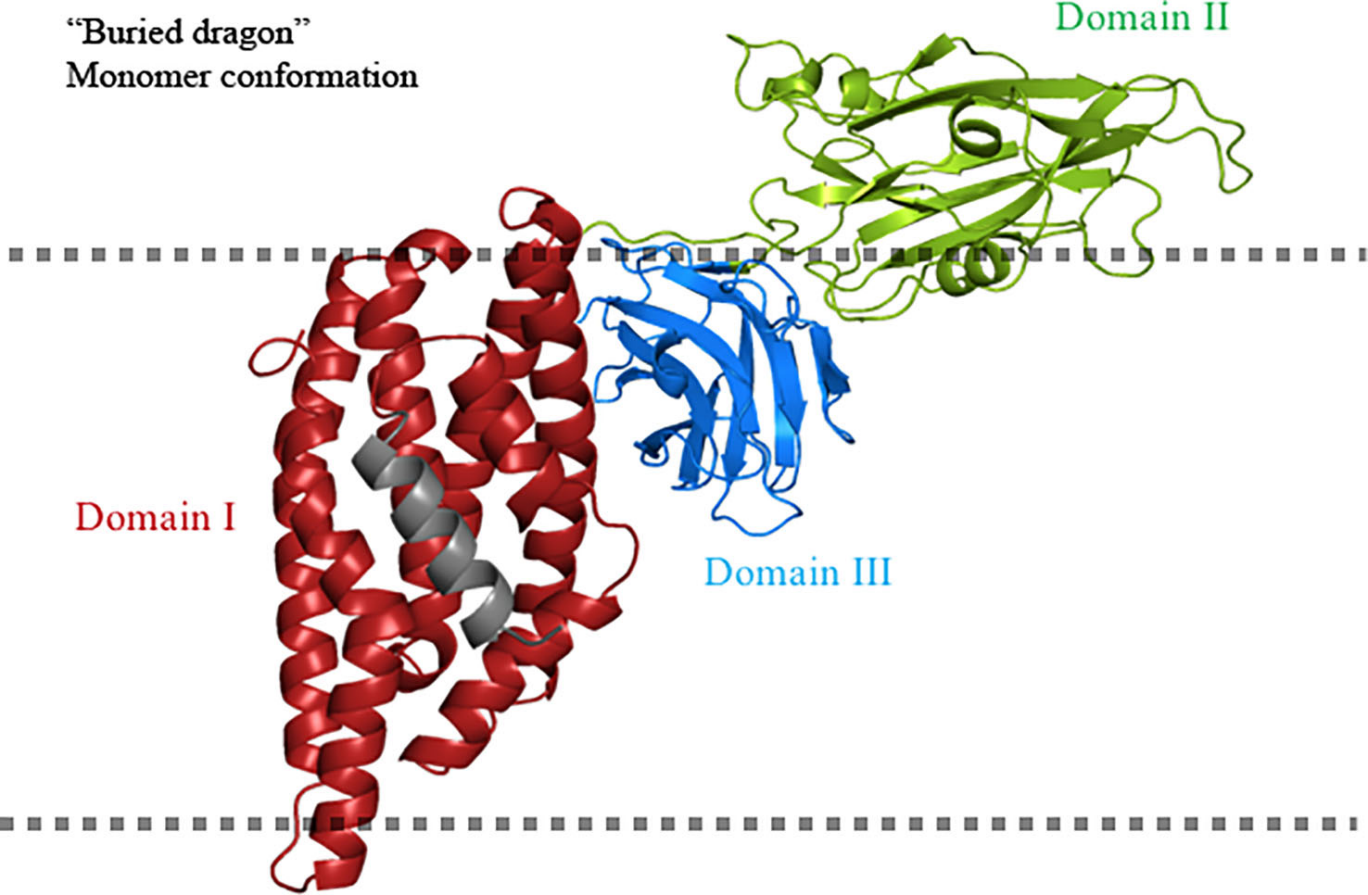
“Buried dragon” model for Cry toxins. Domain I of the Cry toxin inserts into the cell membrane. Domain II is solvent exposed and domain III is buried. The fate of helix α-1 is unclear in this model and is shown in gray. The cell membrane is indicated by the dotted line. The figure was generated by using the PyMol program.

Additional extensive pronase protease digestion analyzes of the Cry1Aa toxin inserted into microvilli membranes isolated from *Bombyx mori*, showed that long regions of the N- and C-terminal ends were protected from protease degradation (54). The processed protein fragments were recognized by different polyclonal antibodies raised against specific regions of the toxin (anti-α2-α3; anti-α4-α5; anti-α6-α7; anti-DII; and anti-DIII). The only region that was not analyzed was helix α-1, as no-antibody was raised to detect this region. The data indicated that, in the soluble activated toxin, domain I is more resistant to proteolysis than domains II and III, indicating a more compact structure of domain I in solution. When the toxin interacts with the microvilli membrane, a relatively large fragment, containing helices α-2 to α-7 and domain III, was protected from protease action. The authors proposed the “buried dragon” model to explain the conformational changes of Cry1Aa when interacting with the target membranes (**Figure 3**). This model proposes that the region containing helices α-2 to α-7 and domain III is buried in the membrane. However, the authors did not recognize that, in addition to membrane insertion, a conformational change in the oligomer structure due to protein–protein contact could also result in protection from proteolysis or antibody detection. Nevertheless, the authors showed that the only region that was not digested and showed similar concentration in all treatments, including those with the lowest and highest protease concentrations, corresponds to a fragment containing helices α-2 and α-3. The authors mentioned that their data indicated that the α-2 and α-3 regions were segregated from the rest of the protein and are likely to be deeply buried in the membrane. In contrast, helices α-4 to α-7 and domain III showed a gradual degradation in the different protease concentrations that were assayed, suggesting that they were more susceptible to protease than helices α-2 and α-3 (54).

### 2.3 The “penknife” model

Based on their analysis of limited proteolysis, electron microscopy, and scanning microcalorimetry assays, Loseva et al. (55) proposed a different conformational model for Cry3Aa toxin when inserted into synthetic liposomes. Electron microscopy data revealed that the Cry3Aa protein had a dramatic effect on the structure of the liposomes, which indicated that the Cry3Aa toxin used in these experiments affected the membrane integrity of the liposomes, implying that it has pore-forming activity.

**Figure 4:**
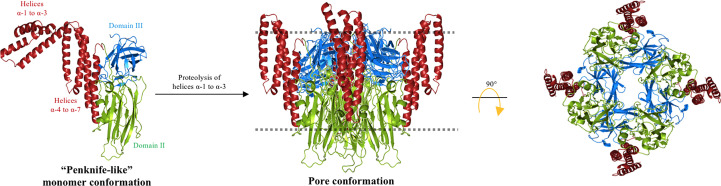
“Penknife” model. Helices α-1 to α-3 of the Cry3Aa toxin is a flexible region that swings away from the core protein and is proposed to be proteolyzed. The rest of the Cry toxin is inserted into the cell membrane (dotted line) forming a pore with domains II and III oriented to the lumen of the pore and the hydrophobic regions of helices α-4 to α-7 are facing the membrane lipids. The figure was generated by using the PyMol program.

Analysis of the temperature dependence in denaturation, enthalpy, and activation energy of Cry3Aa bound to the liposomes showed that this protein undergoes essential conformational changes in the N-terminal end region, from helices α-1 to α-3, whereas the “core protein structure”, consisting of helix α-4 to the C-terminal end region of Cry3Aa, showed no conformational changes upon membrane insertion. A new structural “penknife” model was therefore proposed, where helices α-1 to α-3 play the role of the “blade” (**Figure 4**). This “penknife” model proposed that these N-terminal helices swing away from the toxin core. It was also shown that interaction with lipid vesicles also increased the susceptibility of Cry3Aa to proteolysis (55). Treatment with different proteases revealed that Cry3Aa was cleaved after helix α-3, and it was proposed that helices α-1 to α-3 are degraded upon membrane insertion (55). Owing to the movement of the N-terminal region, the hydrophobic surfaces of helices α-4, α-5, and α-7 would be free to make contact with the membrane lipids, therefore facilitating insertion of the whole core protein into the membrane. The authors proposed that in this arrangement, the pore lumen would be lined by the hydrophilic residues that are present in domains II and III, without detailing which regions of these domains may be facing the pore lumen (55).

However, it is important to mention that it was previously shown that the cleavage of Cry3A by chymotrypsin did not affect the insecticidal activity of Cry3Aa, as the resulting fragments after proteolysis (49, 11, and 6 kDa) remain associated with the core protein under non-denaturing conditions, suggesting that cleavage of this toxin does not result in the proteolytical degradation of helices α-1 to α-3 (56).

This model contradicts the “umbrella” model, as, in the former, it was shown that helices α-4 and α-5 do not change conformation upon membrane insertion. These data support the “buried dragon” model as it was proposed that an important part of the protein is inserted into the membrane, and membrane insertion involves an important movement of the region containing helices α-1 to α-3.

### 2.4 The “folding cane” model

**Figure 5:**
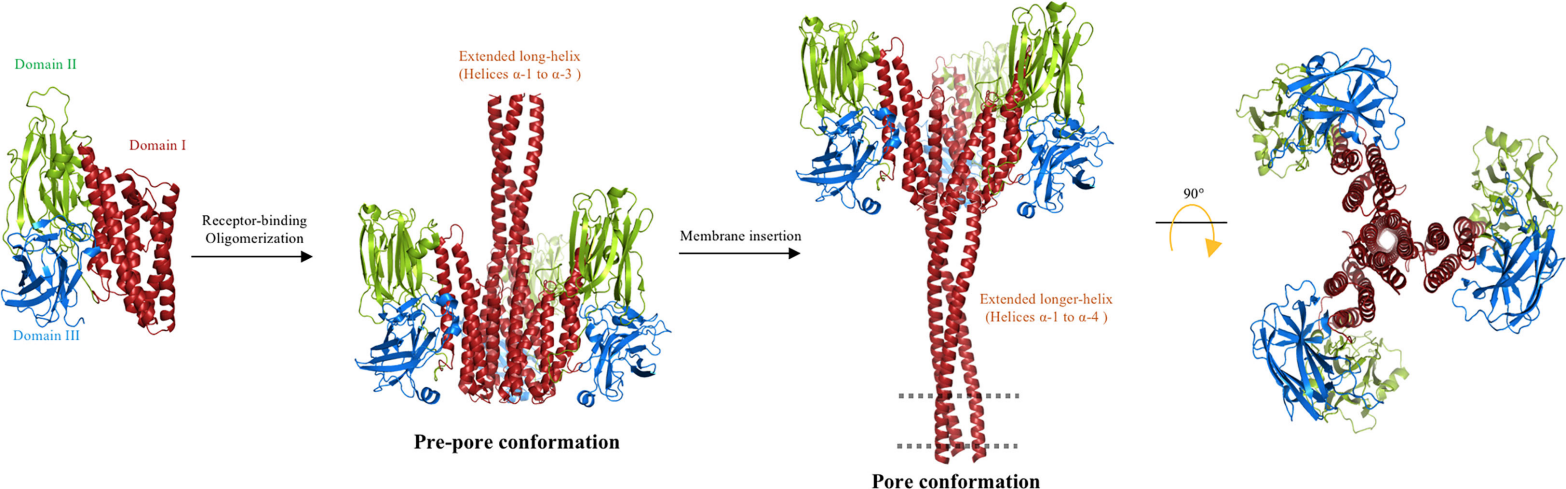
Multistep “folding cane” model. The trypsin-activated Cry toxin binds to membrane receptors, triggering rearrangements at the N-terminal helices of domain I. Helices α-1 to α-3 form a single extended helix that exposes key residues to establish an intermolecular interaction for oligomerization to form a pre-pore. The extended helix requires further conformational changes to form a longer extended helix composed of helices α-1 to α-4 during insertion into the membrane. The region that is proposed to be embedded into the cell membrane (dotted line) corresponds to helices α-1 and α-2a. In agreement with the “umbrella” model, the upper region of the lumen pore is limited by helix α-4, and in this “folding cane” model the pore is along the structure of the extended-helix α-1 to α-4. The long distance between domains II and III and the membrane plane is potentially occupied by protein receptors. The proposed oligomer is composed of three or four Cry toxin subunits. The figure was generated by using the PyMol program.

When the 3D structures of Cry4Ba and Cry5Ba were reported, the authors showed that these proteins were cleaved at the N- terminal end, and that helices α-1 and α-2a were lost during the crystallization process of these proteins. In addition, an extended α-3 helix was formed, by rearrangement of the loop region that connects helix α-2b with helix α-3 and part of helix α-2b (PDB: 1W99 and 4D8M). It is surprising that the cleavage sites were equivalent in Cry4Ba and Cry5Ba, as they were located 50 residues upstream from the end of helix α-3. Moreover, both proteins exhibited a similar trimeric arrangement, where the extended α-3 helix plays a fundamental role in maintaining the trimeric organization, as it displays multiple contacts with other α-helices from adjacent monomers (26, 57). A 3D-model structure of Cry1Ab was constructed based on the coordinates of Cry4Ba and Cry5Ba trimeric arrays. In this Cry1Ab model structure, it was shown that the residue Arg99, which was shown to be involved in oligomerization, was localized in front of an Asp101 residue from an adjacent monomer (58). The proximity of these two charged residues suggests that a salt bridge may have been formed. The study of Cry1Ab mutations, such as the mutant Asp101, supported this assumption, as this Cry1AbD101R mutant was unable to oligomerize and showed a complete loss of toxicity against *M. sexta*, similar to the phenotype of the Cry1AbR99E mutant (48, 58). Interestingly, Cry1AbR99E-D101R, a double mutant that reversed the charges of these two residues, caused Arg99 to change into Glu, and Asp101 to change into Arg and recovered the toxicity and oligomerization of the Cry1Ab protein (58), supporting the idea that this salt bridge is important for Cry1Ab oligomerization and toxicity. This salt bridge is not present in the 3D structure of Cry4Ba or Cry5Ba, but an additional salt bridge in the structure of Cry5Ba (specifically, between the residues Asp129 and Lys131), that links two α-3 helices from adjacent monomers was identified. This Cry5Ba salt bridge is located on the same side of the α-3 helix as the Cry1Ab-salt bridge mentioned above (between R99 and D101). A sequence analysis of the Cry toxin family revealed that the salt bridge identified in Cry1Ab is only partially conserved, as it is found in 24 of the 91 sequences analyzed, whereas the salt bridge identified in Cry5Ba is more conserved, as it is found in 48 of the 91 Cry toxin sequences (58). The salt bridge between the different monomers of Cry5Ba can be formed in other Cry proteins only if a conformational change is produced and an extended helix α-3 is formed in these proteins. Single point mutations in the residues Asp129 and Lys131 of Cry5Ba, and also in Cry1Fa (Cry1FaR85E and Cry1FaE83R), were affected in toxicity, whereas the reverse-charge double mutant in Cry1Fa (Cry1Fa-E83R-R85E) recovered toxicity, supporting that this salt bridge may also be important for stabilizing the oligomeric structure. The Cry1Fa residues, Arg85 and Glu83, are located in the loop between helices α-2b and α-3, thus it was proposed that a conformational change in this region should take place, forming an extended helix α-3, similar to that seen in the Cry5Ba and Cry4Ba proteins. It has been proposed that an extended helix α-3 is required for oligomerization. To test this hypothesis, a series of Cry1Ab double mutants were generated, where two cysteine residues were introduced with the aim of forming disulfide bridges among all the helices that are present in domain I to restrict their mobility. The results showed that all single Cys mutants were active in a way similar to that of the wild-type protein, whereas the double mutant Cry1AbW73C-I97C, which links the Cry1Ab helices α-2b and α-3, was severely affected in terms of oligomerization and toxicity against *M. sexta* and *P. xylostella* larvae, suggesting that helices α-2b and α-3 must undergo a conformational change, supporting that they may form an extended α-3 helix during the toxic activity of Cry1Ab (58). Similarly, double mutations in the Cry1Ab residues Val61 and Ala111, located in helices α-2a and α-3, respectively (Cry1AbV61C-A111C), resulted in a protein severely affected in oligomerization and toxicity against *M. sexta* larvae (59). Finally, a double mutant that links helices α-3 with α-4 at their loop regions, that is loop α-2b/α-3 with the loop α-4/α-5 (Cry1AbI88C-Y153C), showed exactly the same phenotype, as it was severely affected in oligomerization and toxicity against *M. sexta* larvae (59). A similar mutant was constructed in the Cry1Aa toxin in a previous study, and it was shown that its pore-forming activity was affected (51); however, its insecticidal activity was not analyzed. In contrast, two different double mutations, which linked helix α-5 with helix α-6 (Cry1AbV162C-A207C and Cry1AbA165C-Y203C), showed no effects in toxicity or oligomerization, retaining similar toxicity as the wild-type protein and supporting that the movement of helix α-5 is not involved in pore formation. A new model, named the “folding cane” model, was proposed to explain the conformational changes of Cry toxins during pore formation (**Figure 5**). This model proposes that conformational changes between helices α-2a, α-2b and α-3, and also between α-3 and α-4, are important for toxicity. It was therefore proposed that an extended α-helix comprising helices α-2 to α-4 may be formed during oligomerization and pore formation.

In order to prove or discard this new hypothesis, which involves the formation of an extended α-helix conformed by helices α-2 to α-4, the distances between different points in Cry1Ab toxin and the membrane plane were measured by FRET closest approach analyses. These studies showed that during interaction with *M. sexta* microvilli membranes, the Cry1Ab helices α-1, α-2a, and α-2b have a relatively closer distance to the membrane plane than the rest of the protein (59). Additional evidence supporting these conformational changes was obtained from KI quenching analysis of different Cys mutants that located all over the toxin were covalently labeled with a fluorescent dye located all over the toxin. The data showed that fluorescently labeled residues in helices α-1, α-2a, and α-2b showed a dramatic change in solvent exposition from the soluble toxin into the membrane-inserted state, adopting a highly buried conformation. These residues showed a much lower quenching by KI when the protein was inserted into the membrane, indicating indicating that these regions display an important conformational change in their membrane-bound states and are not exposed to solvent (59).

These data indicate that the N-terminal region of Cry proteins, comprising helices α-1, α-2a, and α-2b, and α-3, showed a major conformational change during the insertion of the toxin into the target membrane. The “folding cane” model (**Figure 5**) proposed the formation of an extended long α-helix that is needed for pore formation. This model contradicts the “umbrella” model as conformational changes in helix α-5 were not observed during the insertion of Cry1Ab into the target membrane. In addition, the data that supported the “buried dragon” and Loseva’s “penknife” models also support the “folding cane” model, as it was shown that the Cry1Aa helices α-1 to α-3 and Cry3Aa proteins display important conformational changes during the insertion of these toxins into the membrane (54, 55). Previous data indicating that helices α-3 and α-4 participate in pore-forming activity (41–44, 46, 47, 49) also support the “folding cane” model.

## 3 Structural changes of Vip3 toxins during pore formation

The vegetative insecticidal proteins (Vip3) are a family of PFTs that are also produced by Bt bacteria and comprise more than 130 members across different strains (5; https://www.bpprc.org/database/). Initially, it was shown that Vip3 was lepidopteran specific. However, activity against other insect targets, such as the cockroach species *Periplaneta americana* and *Blattella germanica* (60), and the mosquito species *Aedes aegypti* (61), has been reported. Unlike Cry toxins, which accumulate in parasporal bodies inside the bacterial sporangium, Vip3 proteins are secreted by the Bt bacterium during the vegetative growth phase, and their production continues during the sporulation growth phase (62). A recent report proposed that nutritional growth conditions influence the secretion of Vip3Aa protein, as this protein was secreted into the medium when the bacteria were grown in a rich nutrient medium, in contrast to a sporulation medium, where the Vip3Aa protein was detected inside vegetative cells and also inside the mother cell compartment during the sporulation phase (63).

The Cry and Vip3 proteins produced by Bt are the most studied and used proteins for insect control in agricultural crops worldwide. Similar to Cry proteins, Vip3A protein has also been expressed in transgenic crops for the control of diverse lepidopteran insect pests. Both protein families (Cry and Vip3) are PFTs that bind to different protein receptors to kill their target insects, but several reports have shown that Cry and Vip3 toxins do not share receptors (64) and that no cross-resistance to Vip3A has been observed in insects that have already developed resistance to Cry toxins (65). Therefore, the combination of Vip3 and Cry toxins is a widely used pyramidal strategy, which enables the effective control of insect pests, and counteracts insect resistance to Cry toxins (66).

**Figure 6:**
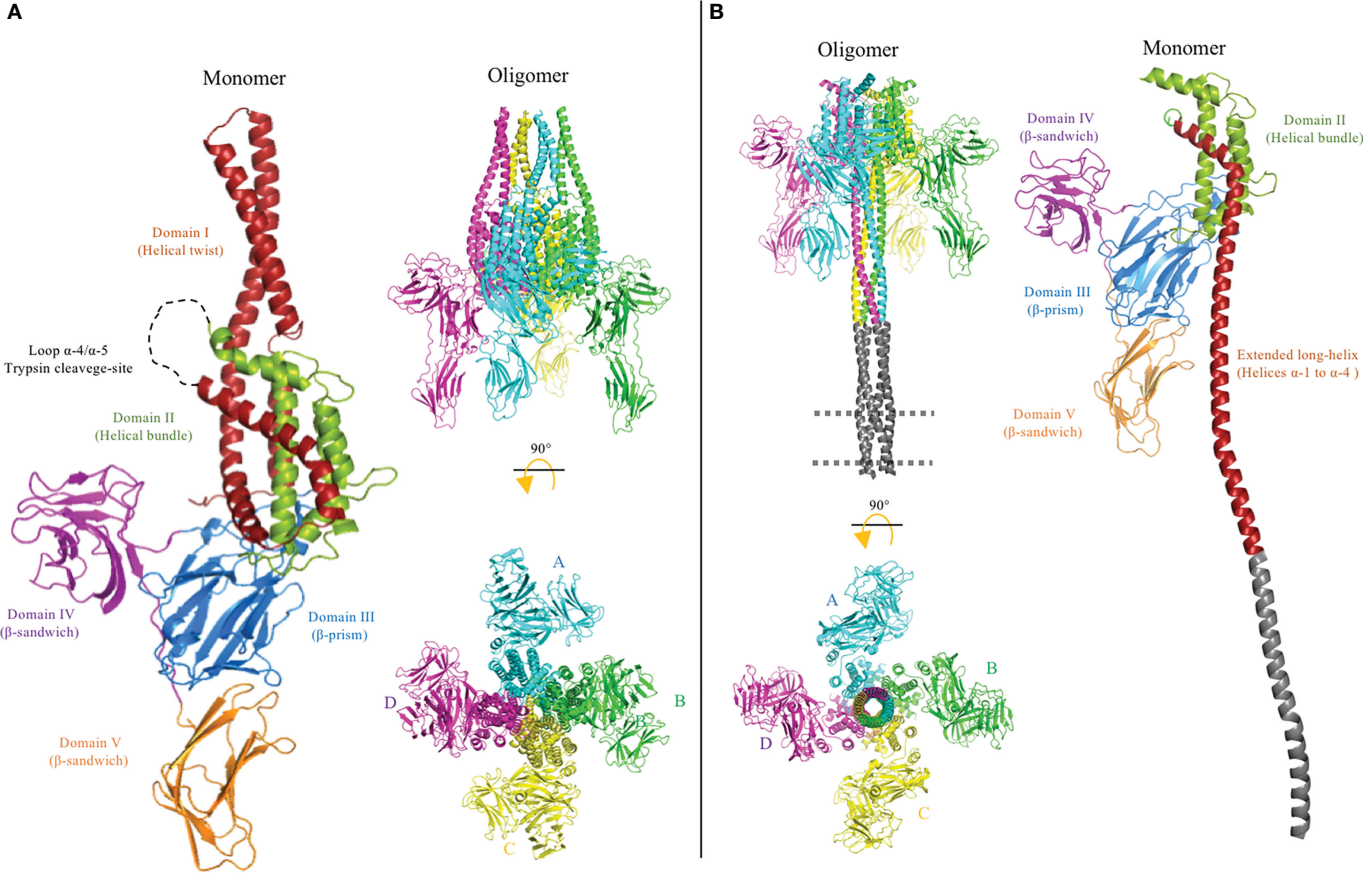
Cryo-electron microscopy (cryo-EM) structures of pre-pore PDB: 6TFK **(A)** and pore PDB: 6TFJ **(B)** of the Vip3 toxin were used to generate this figure by using the PyMol program. The monomer and oligomer of the Vip3 toxin from each conformation are shown. The monomers contain five structural domains, indicated using different colors. Domains are defined according to Núñez-Ramírez et al. (12) and the trypsin cleavage site between helices α-4 and α-5 **(A)** is shown as a dotted loop. After activation, the N-terminal region forms a long four-helical coiled-coil helix needle at the base of the oligomeric complex that is needed for insertion into the membrane and pore formation. It was proposed that the tip of the extended long helix composed of helices α-1 and α-2 (residues 1–94) is inserted into the membrane to form the pore conformation, but these regions were not resolved in the cryo-EM 3D structure. A model of this region is shown in gray. The long distance between the core of the protein and membrane plane is proposed to be occupied by protein receptors.

The Vip3 proteins are composed of approximately 800 residues with a molecular mass of approximately 85–90 kDa, and it has been shown that these protoxins adopt a quaternary oligomeric structure in solution that is composed of four monomers (**Figure 6A**). The mechanism of action of Vip3 proteins also occurs in the insect midgut. After protoxin ingestion the homotetrameric Vip3 protoxin is cleaved by trypsin-like proteases present in the midgut to form an active Vip3 toxin. The cleavage products are two fragments, that is, the N-terminal fragment with a mass of approximately 25 kDa and the C-terminal fragment with a mass of approximately 65 kDa, which remain non-covalently bonded so as to exert insecticidal activity (67). It is recognized that the high specificity of these proteins resides in the receptor interactions that may take place on the microvilli of midgut cells. Currently, it is uncertain whether proteolytic activation occurs before or after receptor binding, or what the identity of the receptor(s) is. Although some Vip3-binding proteins have already been reported, the functional role of these Vip3-binding proteins as receptors is unclear. Ribosome S2 protein (R-S2) (68), fibroblast growth factor receptor protein (FGFR) (69), and scavenger receptor class C protein (SR-C) (70) were identified as putative receptors in the Sf9 cell line that was derived from pupal ovarian tissue of *Spodoptera frugiperda*. Further knock-down assays of these proteins in insect larvae, performed by silencing experiments with RNA interference (RNAi), showed partially reduced sensitivity to Vip3 toxins in *Spodoptera litura*, *S. frugiperda*, and *Spodoptera exigua* (68–70). However, the clustered regularly interspaced short palindromic repeats (CRISPR)/CRISPR-associated protein 9 (Cas9)-mediated knock-out of FGFR and SR-C in *S. frugiperda* revealed that the aforementioned proteins were not functioning as receptors for Vip3Aa toxin as the susceptibility to Vip3Aa did not change in knocked-out (KO) mutant insects (71).

Once the Vip3 is trypsin activated and located on the plasma membrane, the tetrameric Vip3 undergoes a conformational change in order to enter the membrane and form a pore (**Figure 6B**). This pore disrupts the gut epithelium, leading to tissue damage, and, eventually, the death of the insect. In the past 3 years, important progress has been made in the gathering of structural information about the conformational changes of Vip3 toxins during their activation and pore formation. The first X-ray structure of the full-length Vip3Bc1 protoxin was resolved at a resolution of 3.2 Å. In this study, prior to crystallization, the Vip3Bc1 sequence was optimized to its mutant form and the mutated protein was named VIP3B-2160 (72). The VIP3B-2160 mutant was also highly active against different lepidopteran insects and its protoxin structure was shown to consist of five domains (**Figure 6A**). The helices α-1, α-4, α-5, α-6, α-8, and the C-terminal region of α-3, form a helix bundle surrounding the central helix α-7, and this region was defined as domain II, whereas the helices α-2 and N-terminal region of α-3 form a protruding α-helical twist that was defined as domain I. It is important to mention that domains I and II were connected by the long helix α-3. Domain III consisted of three β-sheets with a “Greek-key” topology. Domains IV and V showed a highly similar “β-sandwich” folding structure, composed of two β-sheets. It is interesting to mention that, despite the primary sequences of domains IV and V not being similar, these two domains showed a high structural homology. Moreover, it was shown that the full-length VIP3B-2160 protoxin is organized as a highly stable quaternary structure composed of four monomers, with most of the intermolecular interactions taking place in the N-terminal helical domains. These domains also showed that the homotetrameric VIP3B-2160 protein has an asymmetric conformation, with “two homodimers”. The overall topology has the appearance of a “pyramid”, where the base is formed by domain II, and the apex by domain I. Intermolecular interactions between the four subunits take place mainly in helices α-4, α-5, and α-6 of domain II, forming a hydrophobic core. The tip of the α-helical twisted domain I has an interface between two subunits from each homodimer, whereas the two other subunits of domain I do not interact with each other and are located at opposite sides of the interface. Domains III–V are oriented outside the pyramid base and are exposed to the solvent (**Figure 6**).

A tetrameric complex was also observed by transmission electron microscopy (TEM) for the Vip3Ag4 protoxin (73), which was consistent with the X-ray structure of VIP3B-2160 protoxin. Interestingly, the activated Vip3Ag4 toxin also preserved a quaternary conformation after trypsin activation, as identified in previous TEM studies (73).

A second X-ray structure of the activated Vip3Aa11 toxin was also resolved at a resolution of 3.2 Å. For its crystallization, the approximately 65 kDa C-terminal region of the Vip3Aa11 toxin (residues 200–789) was fused to a maltose-binding protein (MBP). The X-ray crystal structure of Vip3Aa11 revealed a monomeric protein consisting of four domains (74). As the approximately 25-kDa N-terminal fragment (residues 1–198) was substituted by the MBP, this region was assumed to form an independent domain. Domain II contains five anti-parallel α-helices arranged in two layers. The α-3, α-4, and α-5 helices retain the same arrangement to equivalent helices α-6b, α-7, and α-8 of the VIP3B-2160 protoxin, whereas the two N-terminal α-helices differ significantly. Domains III–V presented a similar folding to the VIP3B-2160 protoxin structure, except that the C-terminal domain V of the toxin bends to adopt a perpendicular orientation relative to domain III. However, the truncated Vip3Aa11 toxin was not active against Sf9 cells or *S. exigua* larvae, which is consistent with the hypothesis that both protein fragments (approximately 25 and 65 kDa) are required for Vip3 toxicity (67). It is highly probable that the truncated Vip3Aa11 toxin adopts a monomeric conformation because it lacks the N-terminal α-helices involved in the intermolecular interactions observed in the VIP3B-2160 protoxin.

Finally, the molecular structures of Vip3Aa16 and Vip3Bc1 protoxins were determined by single particle cryo-electron microscopy (cryo-EM) analyses performed at a 2.9 Å and 3.90 Å global resolution, respectively (12, 13). The recorded topology of these two Vip3 protoxins resemble the VIP3B-2160 quaternary structure, also consisting of five domains organized as a homotetramer complex (**Figure 6B**). Núñez-Ramírez et al. (12) defined the N-terminal helices from α-1 to α-4 as domain I, according to the trypsin cleavage site located in residue Lys198 that resulted in the production of two approximately 65- and 25-kDa fragments upon protoxin activation (12), whereas Byrne et al. conserved the nomenclature from the VIP3B-2160 protoxin, which defined the protruding α-helical twist α-2/α-3 as domain I (13). Regarding the C-terminal end, the β-strand domains III–V of Vip3Aa16 and Vip3Bc1 showed similar folding to the X-ray structure of Vip3Aa11 and VIP3B-2160. Notably, both approaches to solving the structures (i.e., cryo-EM and X-ray analysis) revealed that the C-terminal domain V of Vip3A protoxins (Vip3Aa11 and Vip3Aa16) and Vip3B protoxins (VIP3B-2160 and Vip3Bc1) show the same orientation, being down-bent in the case of Vip3B protoxins. All these data together show a highly reliable structure of homotetrameric Vip3 protoxins in solution, with the exception of the X-ray structure of the truncated Vip3Aa11, which presented a monomeric conformation given that the N-terminal helices of domains I and II were substituted by MBP, preventing its oligomerization.

As previously mentioned, the surface topology of the trypsin-activated Vip3Ag4 toxin analyzed by TEM showed a highly stable homotetramer (73). This result was confirmed by the analysis of the cryo-EM structure of the trypsin-activated Vip3Aa16 (12) and Vip3Bc1 (13) resolved to a 2.9 Å and 4.8 Å global resolution, respectively. However, these cryo-EM structures revealed that the architecture of both homotetrameric Vip3 protoxins undergo a major conformational change at the α-helices of the N-terminal end upon trypsin activation. In particular, the pyramid apex composed of helices α-1 to α-4 turned downward, and were remodeled as a single long α-helix. A coiled-coil structure of four extended helices form a needle approximately 200 Å long. It was proposed that large conformational changes of helices α-1 to α-4 are enabled through intermolecular grooves flanked by the three β-stranded domains. However, the cryo-EM structures of trypsin-activated Vip3 toxins show poor electron density for helices α-1 and α-2, and for this reason it was not possible to obtain structural coordinates from the distal region of the helical needle (12, 13). The ordered upper region of the needle forms a tunnel with a radius of up to 2 Å. The trypsin-activated Vip3Bc1protein was associated with a synthetic liposome by the coiled-coil needle, and it was proposed that the tip of this helical bundle is the region that penetrates into the membrane (13). On the other hand, the three C-terminal domains III-V of trypsin-activated Vip3 toxins remain unchanged (12, 13).

The trypsin cleavage-site of the Vip3 toxins is located in the connecting loop between helices α-4 and α-5. Among different members of the Vip3 toxin family, this loop is solvent exposed in the homotetramer conformation and frequently contains positively charged residues, such as lysine, suggesting a conserved molecular mechanism for triggering conformational changes, leading to the activation and pore formation of Vip3 proteins.

It is noteworthy that, although Cry and Vip3 proteins have different primary sequences, the structural domains of Vip3 toxins resemble the first three domains reported for Cry toxins. Aside from the common shape-folded domains, it has been widely accepted that β-stranded domains of Vip3 and Cry toxins are involved in receptor binding (75), whereas the helical domains play a critical role in oligomerization and membrane insertion (76). The α-helical domain I of Vip3 is not present in Cry toxins, and Vip3 has two β-sandwich structural domains (domains IV and V) that share structural similarities with the β-sandwich domain III of Cry toxins. Domain II of Vip3 proteins shares a similar protein fold with domain I of Cry proteins, whereas domain III of Vip3, composed by antiparallel β-sheets forming a β-prism fold, is structurally similar to domain II from Cry proteins (12, 13). These features suggest a strongly convergent evolution of the insecticidal PFTs produced by Bt bacteria. The “folded cane” model of pore formation of Cry proteins resembles the conformational changes shown by Vip3 proteins after activation, suggesting that a similar mechanism could be involved in the pore formation of both proteins, where an important conformational change is observed in helices located at N-terminal regions.

## 4 Structural changes of Tc toxins during pore formation

Tc toxins are a family of large, multi-subunit protein complexes produced by certain bacterial strains, such as *P. luminescens* and *X. nematophila* (4). The Tc toxin complex typically consists of three components: the TcA, TcB, and TcC proteins. These toxin components are encoded in a genomic region known as the *tc* locus and play an important role in the pathogenesis of these bacteria and the killing of their host organisms, which include insects and other invertebrates. Tc toxins are important in the symbiotic relationship between these bacteria and nematodes from the family Heterorhabditidae. The nematodes use the Tc toxins produced by their symbiotic bacteria to kill the target insect and create a nutrient-rich environment where the nematode can grow and reproduce. However, Tc toxins have been reported to also be present in other entomopathogenic bacteria, such as *Serratia entomophila* (77), *Yersinia entomophaga* (78), and *B. thuringiensis* (79, 80). In fact, the Tc toxins are widely distributed within the Bacteria domain, including in human pathogenic bacterial strains, such as *Salmonella*; however, their functional role remains unknown (81).

Studies on Tc toxins from Bt bacteria are scarce; most information regarding these toxins has been derived from *P. luminescens* and *X. nematophila*. However, genomic analyses have confirmed the prevalence of the *tc* locus in several Bt strains (http://www.mgc.ac.cn/dbTC). The influence of the Tc toxin on the insecticidal activity of the Bt IBL90 strain show that the transcription of the TcA component was activated when *Lymantria dispar* larvae ingested this bacterium, suggesting that Tc toxins may play a critical role in insect pathogenesis (79). Interestingly, the *tc* locus of the Bt strain GR007 is located on a large plasmid, which also contains *cry* and *vip* genes (80). These data suggest that it is probable that the bacterium Bt has acquired the *tc* locus by horizontal transfer from other bacteria that share common ecological niches, such as soil or insects. In addition, the genetic organization of the *tc* locus is highly conserved among different Bt strains, suggesting that the acquisition of Tc toxins was a recent event. This organization consists of a single *tc* locus harboring the TcA component divided into two open reading frames (*tcaA* and *tcaB*), a single TcB component (*tcbC*), and two TcC components (*tccC1* and *tccC2*). Among different bacteria that contain the TcA component, this gene is frequently encoded in two ORFs, which are required to form the functional full-sized TcA component (81).

**Figure 7:**
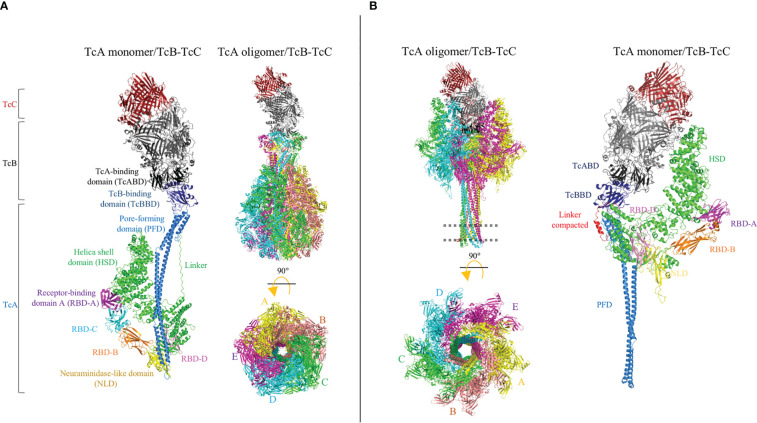
Cryo-electron microscopy (cryo-EM) structures of the Tc holotoxin from *Photorhabdus luminescens*. The tripartite complex comprising TcA (TcdA1), TcB (TcdB2), and TcC (TccC3) from *P. luminescens* is shown before (pre-pore, **(A)** and after (pore, **(B)** membrane insertion. The Tc holotoxin contains five subunits of the TcA component, forming a bell-shaped oligomer, and the TcB–TcC cocoon complex is positioned on the upper region of the TcA oligomer. Note that the linker region is partially structured in the pore conformation (red). The cell membrane is indicated by a dotted line. Figures were prepared from deposited cryo-EM structures of the Tc complex from *P. luminescens*, PDB:6H6E and 6SUF, by using the PyMol program.

The Tc toxin complex is a large heptameric structure of approximately ≈ 1.7 MDa, which contains the three Tc components (TcA, TcB and TcC) in a molar ratio of 5 : 1 : 1 (**Figure 7A**). The TcA component is a large protein of approximately 280 kDa and forms a homopentameric complex resembling a bell. Each TcA monomer contains eight domains: the N-terminal region of TcA contains the helical shell domain (HSD), four receptor-binding domains (RBD), and a neuraminidase-like domain (NLD), whereas the C-terminal region of TcA contains the TcB-binding domain (TcBBD) and a pore-forming domain (PFD). The N-terminal and C-terminal regions of this TcA monomeric structure are connected by a linker. The heptamerical complex contains a pore-forming helical needle of approximately 200 Å composed of five PFDs from each TcA subunit. The needle is surrounded by a solvent-exposed shell composed of HSDs, RBDs, and NLDs (82). The TcB and TcC components form a heterodimer complex of approximately 280 kDa that is coupled to the upper region of the bell-shaped complex. The TcB–TcC dimer is a β-barrel “cocoon” that encloses the C-terminal hypervariable region (HVR) of the TcC protein in a chamber. Binding between the large TcA pentameric complex and the TcB–TcC dimer is enabled by the β-propeller TcA-binding domains of TcB (TcABD) and TcBBD (83).

Once the complete Tc complex is assembled, it is proposed that the RBD of TcA interacts with the host cell membranes. Unlike Cry and Vip toxins, the Tc toxin complex has been described as possibly having cytotoxicity against mammalian cells and insect pests. The promiscuous activity of the Tc complex is probably due to the fact that the Tc toxin recognizes cell surface glycans (81, 84). Recently, the Visgun (Vsg) protein was identified as the first protein receptor, and its functional role was proven in mammalian U2OS cells expressing Vsg, orthologous from dipteran, coleopteran, and lepidopteran insects (85). However, it was also proven that glycosylation of the Vsg protein may modulate the specificity of Tc toxins. In either case, whether by glycan- or protein-mediated recognition, the Tc complex is finally internalized into the cell *via* endocytosis and a large conformational change is required to form the pore (**Figure 7B**). The bell-shaped TcA complex opens a tiny hole, at the shell bottom, and the inner helical needle moves downward to penetrate the membrane, forming a pore (14). Contraction of the linker region showed that the downward drive of the helical needle plays a pivotal role, where the linker is compacted and partially folded as an α-helix of the pore. Concomitantly, the TcBBD–TcABD interface opens a gate, forming a continuous pore between the TcA needle and TcB–TcC chamber. Then, the aspartyl protease domain from the TcC component cleaves its C-terminal HVR domain, which translocates into the cytoplasm of host cell across the pore (86). Once inside, the HVR disrupts the normal function of the cell, leading to cell death. The HVR components TccC3 and TccC5 showed adenosine diphosphate (ADP)-ribosyltransferase activity in actin and RhoA proteins, respectively (15). Although it has been observed that the TcA component is sufficient to cause cell damage by pore formation, the addition of the TcB–TcC complex blocks the pore and the cytotoxic effect is significantly enhanced (87).

## 5 Final conclusions

Bt bacteria have become specialized to kill insect pests by expressing several PFTs. These bacteria have incorporated the codifying sequences of multiple insecticidal PFT proteins into their genome. A special organization of these genes in pathological islands containing different genes that encode the insecticidal PFTs, that show toxicity against insects from the same order (either lepidopteran, coleopteran or dipteran) have been described in multiple Bt strains, where *cry* genes may be grouped with *vip* and *tc* genes (80, 88).

It is surprising that the 3D structure of Vip3A shows important similarities with the structure of the Cry toxin, suggesting that these two proteins may have similar mechanisms of pore formation (12, 13). However, these toxins differ in their specificity, because they have different interactions with receptor proteins in susceptible insect pests (64–66). It was proposed that the Cry protein may exhibit similar conformational changes to the Vip3A toxin when inserted into the membrane, where an extended α-helix in the N-terminal region is formed during the pore-forming activity of both PFTs. (12, 13, 58, 59). This hypothesis still remains to be proven by structural studies of Cry proteins. Interestingly, other α-PFTs have shown an α-helical needle spanning the membrane with a flexible tip involved in pore formation (3, 89), suggesting that this may be a general strategy of PFTs to span the membrane and attack their specific targets.

Overall, the data supporting the “buried dragon”, Loseva’s “penknife” and “folding cane” models contradict the “umbrella” model. Of all these models that describe the conformational changes of Cry toxins during their insertion into the membrane, the “folding cane” model best explains the previously reported data, including those that supported the “buried dragon” and Loseva’s “penknife” models (54, 55). In addition, the data supporting the role of helices α-3 and α-4 in oligomerization and pore-forming activity support the “folding cane” model (46, 47, 49), as the “folding cane” model proposes that major conformational changes in the N-terminal region of Cry toxins, specifically those involving helices α-1 to α-3, are required to form the pore together with helix α-4.

**Figure 8:**
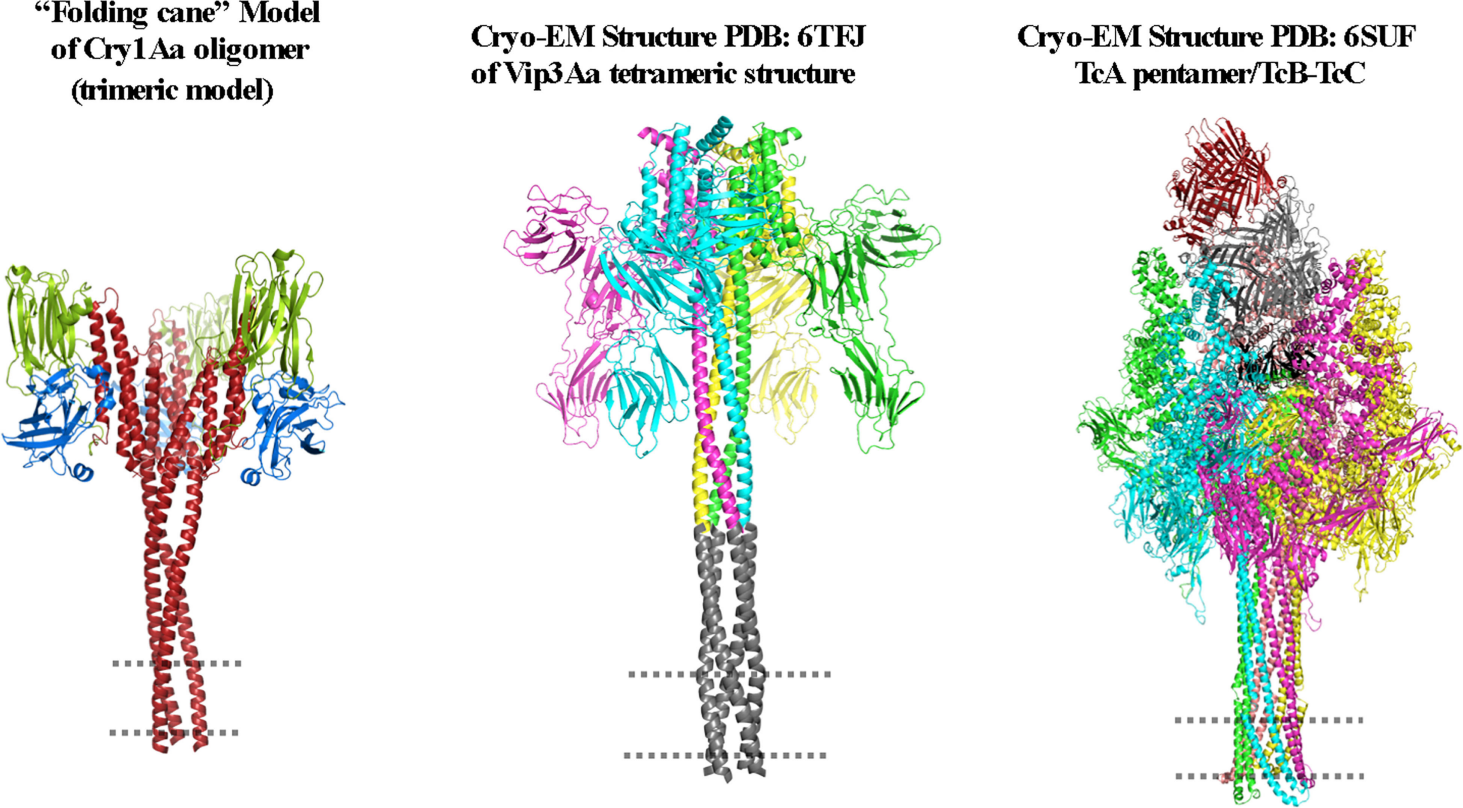
Comparison of the proposed structures of the Cry, Vip3, and Tc toxins when the oligomeric structures of these toxins are inserted into the membrane. The figure was generated by using the PyMol program.

**Figure 8** shows that Cry, Vip3 and Tc toxins undergo major structural rearrangements, producing an extended α-helix that plays a fundamental role in perforating their target membranes and inducing pore-forming activity. It is important to note that all of these proteins can be expressed in the same Bt strain. In addition, the structural similarities between Vip3 and Cry proteins, in terms of their 3D structures and proposed mechanisms of pore formation, suggest that both proteins may share a common evolutionary origin. Therefore, further studies should focus on analyzing the evolutionary processes of these two important protein families. In addition, it is crucial to define the proteins that act as receptors for Vip3 in the different target insect pests. The structural and functional similarities of Vip3 and Cry toxins may also indicate that it could be possible that cross-resistance, based on mechanisms other than receptor binding, could evolve in target insects. However, this is merely speculation and further studies are required. Finally, the mechanisms associated with Vip3A resistance will also need to be described. Recent data suggest that the mechanisms of insect resistance to Vip3A proteins are different from those described in insects that have evolved resistance to Cry proteins: it was reported that in some insects, such as *Mythimna separata*, the resistance to Vip3Aa is not related to altered binding to microvilli membranes (90). Furthermore, reduced expression of ALP in a Vip3-resistant strain of *Heliothis virescens* did not affect the binding of Vip3Aa to the microvilli membrane (91).

## Author contributions

SP and AB participated in writing the first draft of the manuscript. All authors contributed to the article and approved the submitted version.
